# Draft Genome Sequence of *Streptomyces* sp. Strain R1, Isolated from Water Canal Sediments, Possessing Antimicrobial and Plant Growth Promoting Capabilities

**DOI:** 10.1128/mra.00725-22

**Published:** 2022-08-16

**Authors:** Neelma Ashraf, Munir Ahmad Anwar, Kalsoom Akhtar, Sumera Yasmin, Shazia Khaliq

**Affiliations:** a Industrial Biotechnology Division, National Institute for Biotechnology and Genetic Engineering (NIBGE), Constituent College of Pakistan Institute of Engineering and Applied Sciences (PIEAS), Faisalabad, Pakistan; b Chemical Ecology/Biological Chemistry, Department of Biology & Zukunftskolleg, University of Konstanz, Konstanz, Germany; University of Southern California

## Abstract

We present the genome sequence of *Streptomyces* sp. strain R1, isolated from water canal sediments and possessing genes responsible for antimicrobial metabolites and plant growth promotion. The genome assembly contains 7,936,694 bp with 72.24% of guanine-cytosine content. This genome will provide basic knowledge of the genes and pathways involved in the above mechanisms.

## ANNOUNCEMENT

*Streptomyces* are soil-inhabiting, free living, Gram-positive filamentous bacteria that also present as symbionts of plants, insects, and animals (1). Due to their production of a broad range of bioactive secondary metabolites, *Streptomyces* have gained attraction in agriculture as biocontrol agents (2–9).

*Streptomyces* sp. strain R1 was isolated from water canal sediments from Faisalabad, Pakistan. 1 g of sample was suspended to serial dilution and cultured on casein starch peptone yeast-extract malt-extract (CSPY-ME) medium for 7 days at 30°C (10). A hard, powdery colony was selected and purified by restreaking onto a fresh plate. For genomic DNA extraction, a 5-day-old culture was subjected to a previously developed method with certain modifications (11). Three stainless steel beads were placed in a culture-containing Eppendorf tube and incubated at 37°C for 25 min after being rinsed with 120 μL of an extraction solution that contained lysozyme (0.1 mg/mL). Proteinase-K (20 mg/mL) and RNase-A (0.1 mg/mL) were added, followed by a 5 min incubation at 65°C, to denature and remove proteins and RNA. The extracted DNA was resuspended in EB buffer with the same volume of solid-phase reversible immobilization (SPRI) beads (Bulldog Bio Inc., USA) for purification. A Quantit dsDNA HS assay (Thermo Fisher Scientific) was performed in triplicate to quantify the purified DNA using an Ependorff AF2200 plate reader (Eppendorf UK Ltd., UK). The Nextera XT Library Preparation Kit (Illumina, San Diego, USA) was employed to prepare the genomic DNA libraries, making several modifications to the manufacturer's protocol due to the high guanine-cytosine content. In the polymerase chain reaction (PCR), 2 ng of DNA were used instead of 1 ng, and the elongation period was lengthened from 30 s to 1 min. For the DNA quantification and library preparation, Microlab STAR liquid handler (Hamilton Bonaduz AG, Switzerland) was used, and the Illumina libraries were created and quantified using a KAPA Library Quantification Kit (Roche LightCycler 96 quantitative PCR). Sequencing with 250 bp paired-end reads was done using an Illumina HiSeq 2500. Default parameters were employed for all specified “software”.

Quality control was checked on pair end reads generating 178× coverage. Trimmomatic 0.30 software was used to trim the adapted reads at a sliding window quality cutoff of Q15 (10, 12). For the variant calling, reads from the samples were separately aligned to references using bwa-mem 7.12 (13) under the default settings. A pileup file was generated from all of the alignments using SAMtools 1.9 (https://sourceforge.net/projects/samtools/files/samtools/1.9/) mpileup, again under the default settings. Variant matching was performed using VarScan (version 2) (http://dkoboldt.github.io/varscan/) mpileup2cns under two different settings (for the two different output files), one with a 10% threshold (min-coverage, 3; min-var-freq, 0.1; *P* value, 0.05) and the other with a 90% threshold (min-coverage, 10; min-var-freq, 0.9; *P* value, 0.05), and this was annotated using snpEff (http://pcingola.github.io/SnpEff/). The *de novo* assembly was made with the help of SPAdes version 3.7 (14), using the default parameters, and contig annotation was carried out with Prokka 1.11 (15). A completeness analysis was performed by using the Microbial Genomes Atlas (MiGA) webserver (http://microbial-genomes.org/). The metrics were assembled using the Prokaryotic Genome Annotation Pipeline (PGAP) version 4.12 by NCBI (https://www.ncbi.nlm.nih.gov/genome/annotation_prok/).

*Streptomyces* sp. strain R1 has a high quality genome with a completeness of 99.1%, a genome size of 7,936,694 bp, a mean coverage of 178×, a number of 3,125,371 reads, and a guanine-cytosine content of 72.24%. The genome assembly produced 113 contigs with an *N*_50_ value of 237,932 bp, with a length of 1,198,146 bp for the largest contig and 6,866 coding sequences with 67 tRNAs and 3 ncRNAs.

### Data accessibility.

The whole-genome sequencing project JAJIBA000000000, BioProject number PRJNA777709, SRA number SRX13087810, and BioSample number SAMN22870856 have all been deposited in GenBank. Furthermore, the accession number OL744553 was assigned for the 16S rRNA sequence in ENA/DDBJ/GenBank.
